# Supplementary figures and images for: Towards remote monitoring in pediatric care and clinical trials—Tolerability, repeatability and reference values of candidate digital endpoints derived from physical activity, heart rate and sleep in healthy children
Source: PLoS One. 2021 Jan 7;16(1):e0244877. doi: 10.1371/journal.pone.0244877 (PMC7790377; doi:10.1371/journal.pone.0244877)

S1 Fig. Median (IQR) compliance to all study tasks by age

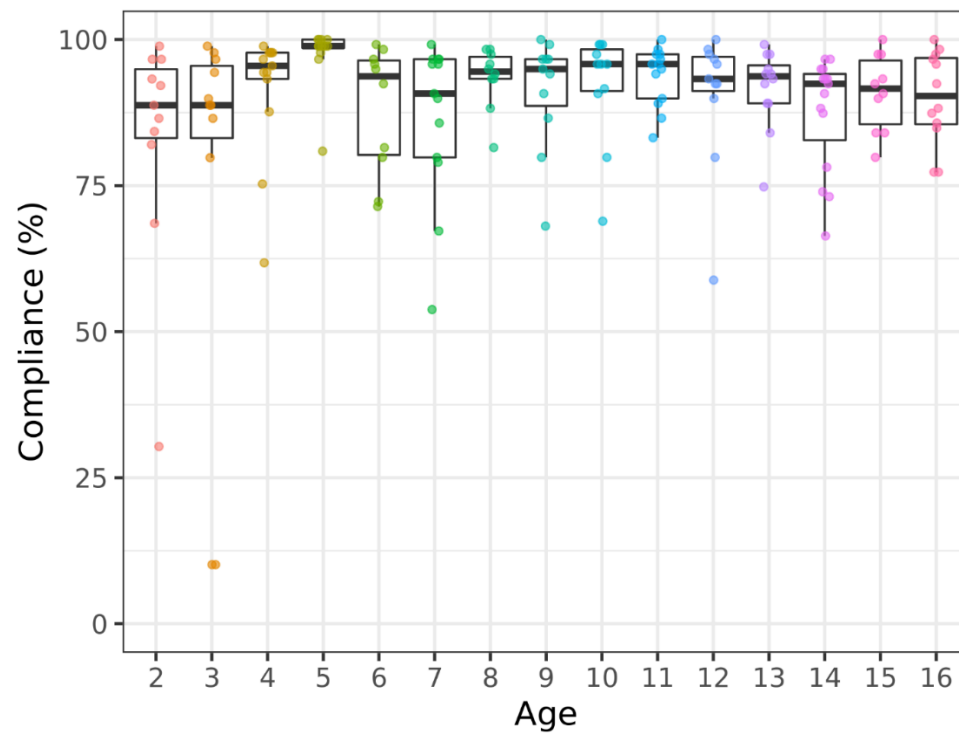

Supplement: S1 Fig — (PDF) [file pone.0244877.s001.pdf]

S2 Fig. Decrease in compliance over time

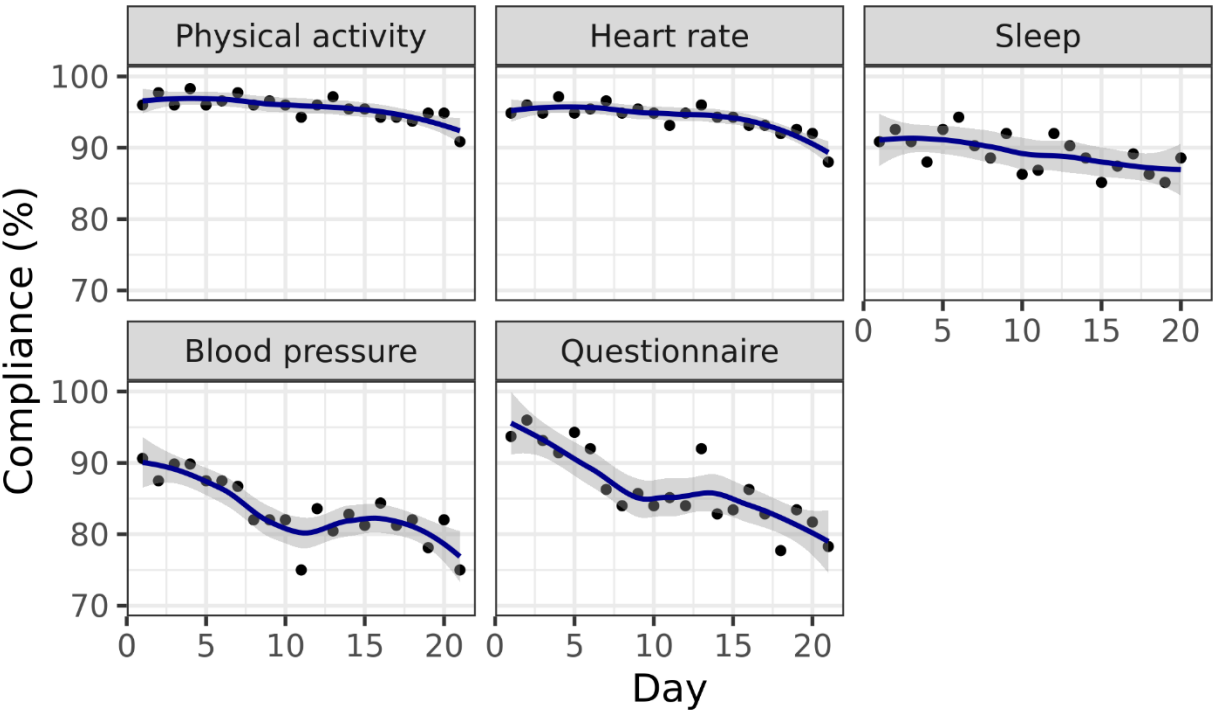

Supplement: S2 Fig — (PDF) [file pone.0244877.s002.pdf]

S4 Fig. Blood pressure measurements per age year

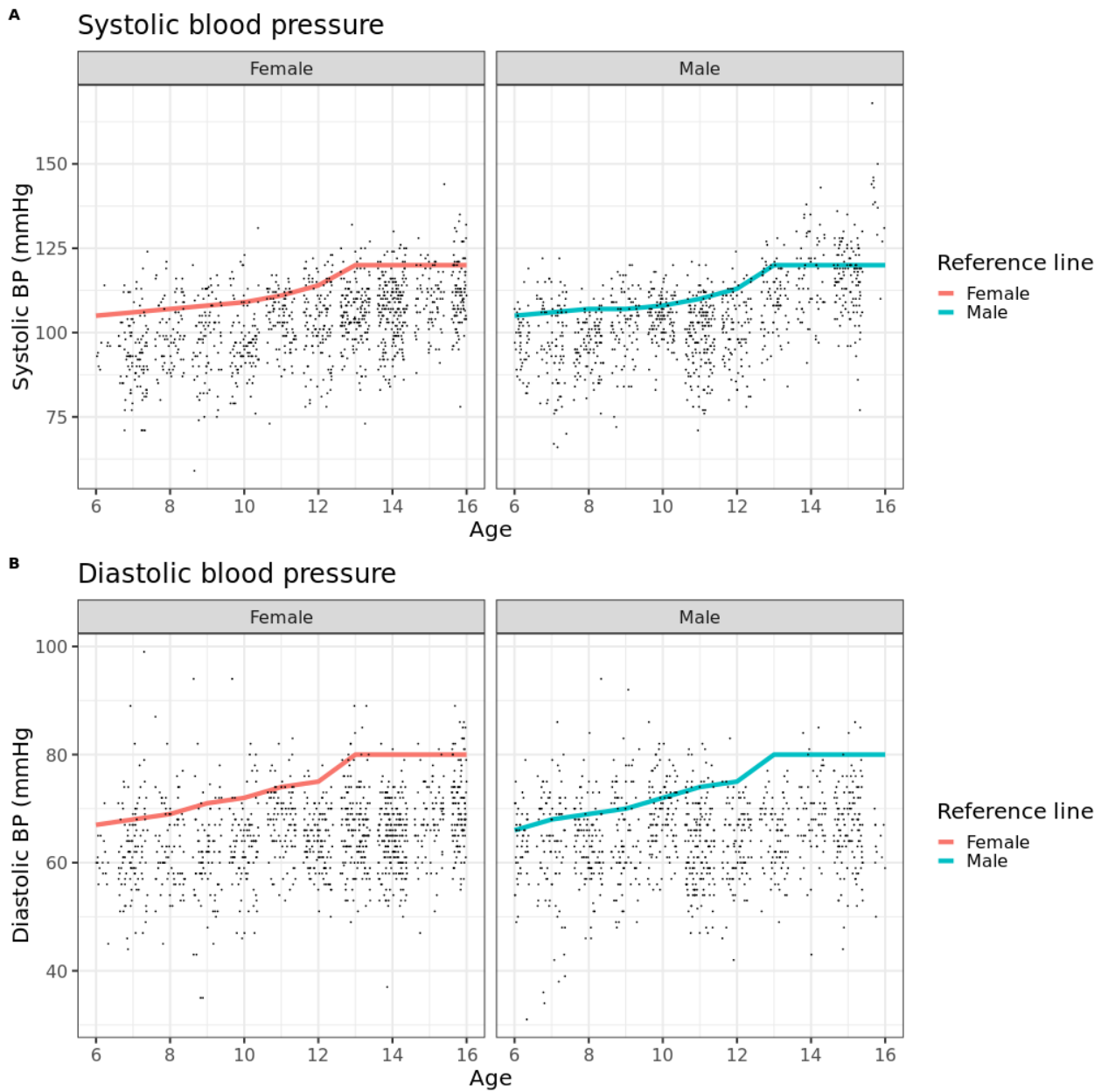

Supplement: S4 Fig — (PDF) [file pone.0244877.s004.pdf]
