# Supplementary material for: Towards remote monitoring in pediatric care and clinical trials—Tolerability, repeatability and reference values of candidate digital endpoints derived from physical activity, heart rate and sleep in healthy children
Source: PLoS One. 2021 Jan 7;16(1):e0244877. doi: 10.1371/journal.pone.0244877 (PMC7790377; doi:10.1371/journal.pone.0244877)
Supplement: S3 Fig — (PDF) [file pone.0244877.s003.pdf]

**S3 Fig. Sleep duration by day of the week**

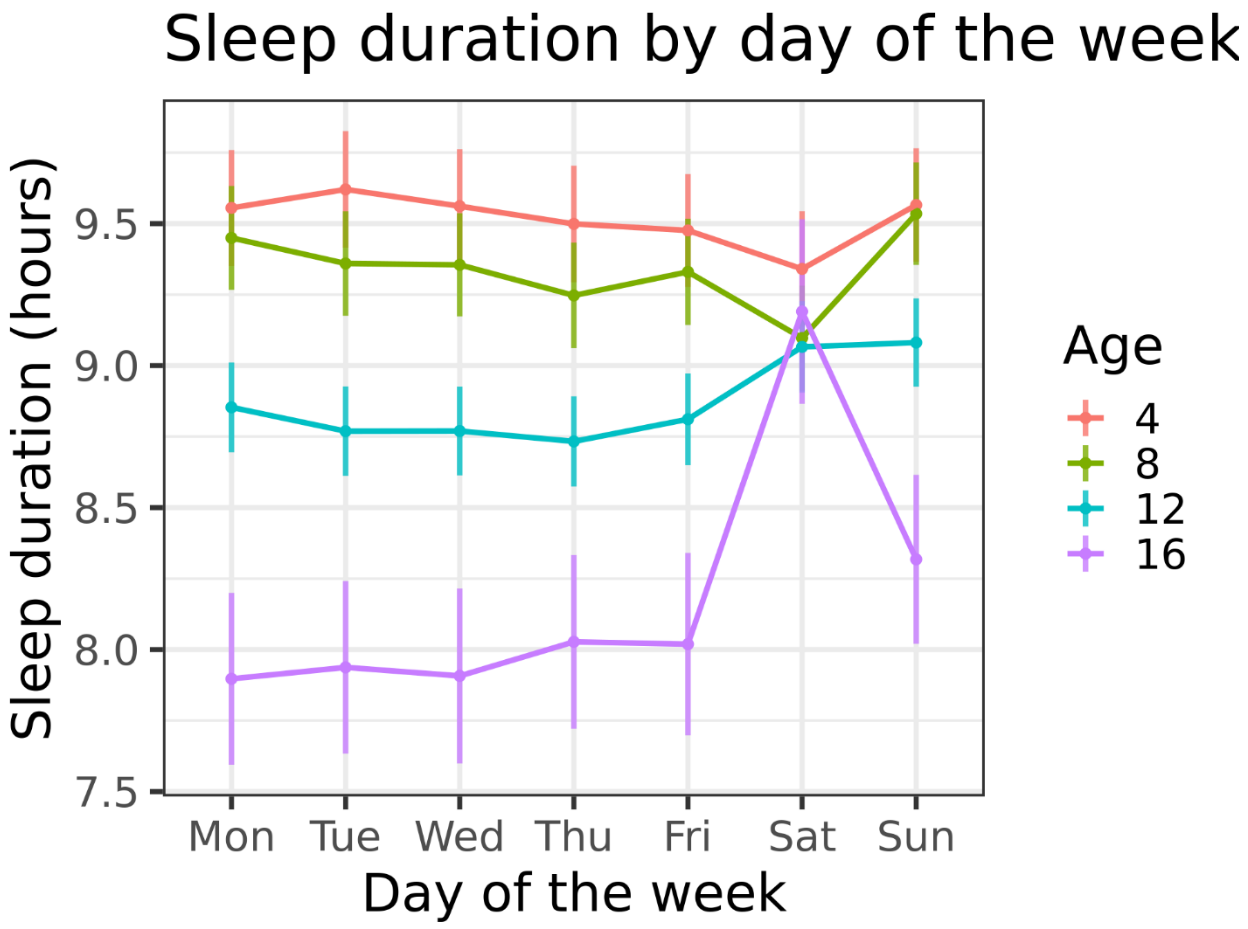


Total sleep duration by day of the week for 4 different ages. A limited amount of ages is displayed to maintain clarity.
