# Supplementary material for: Towards remote monitoring in pediatric care and clinical trials—Tolerability, repeatability and reference values of candidate digital endpoints derived from physical activity, heart rate and sleep in healthy children
Source: PLoS One. 2021 Jan 7;16(1):e0244877. doi: 10.1371/journal.pone.0244877 (PMC7790377; doi:10.1371/journal.pone.0244877)
Supplement: S5 Fig — (PDF) [file pone.0244877.s005.pdf]

S5 Fig. Temperature measurements per age year

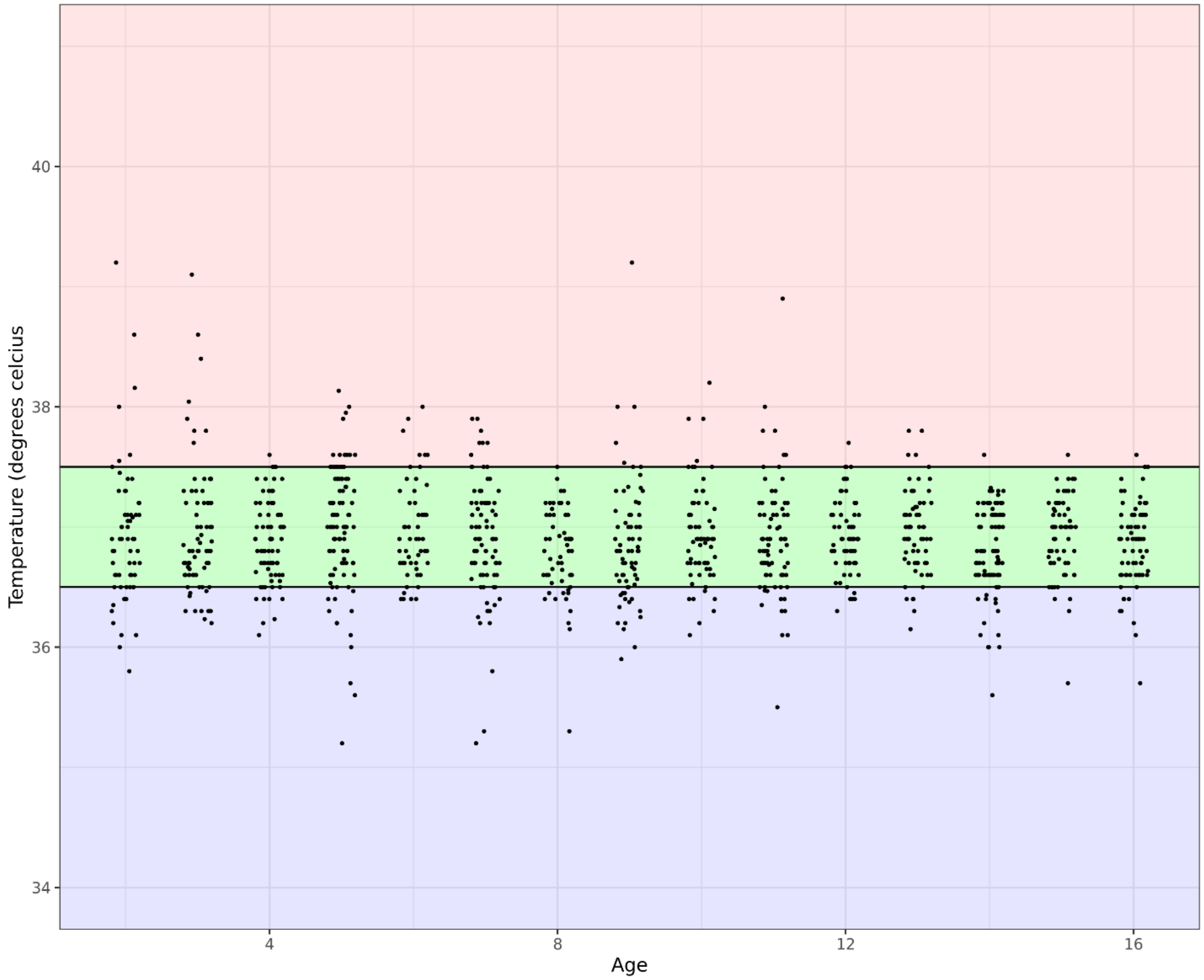

Graphical representation of temperature measurements during the study. Green area represents 36.5-37.5 °C. Red and blue area indicates a temperature above or below this range, respectively.
