# Supplementary material for: Towards remote monitoring in pediatric care and clinical trials—Tolerability, repeatability and reference values of candidate digital endpoints derived from physical activity, heart rate and sleep in healthy children
Source: PLoS One. 2021 Jan 7;16(1):e0244877. doi: 10.1371/journal.pone.0244877 (PMC7790377; doi:10.1371/journal.pone.0244877)
Supplement: S1 Table — (PDF) [file pone.0244877.s006.pdf]

S1 Table. Tolerability questionnaire responses

Question	Agreement with statement (Mean (SD)) (n =160)
**My child liked wearing the watch**	4.4 (0.9) out of 5
**My child is happy the study is over**	2.7 (1.3) out of 5
**My child moved more than usual during the study**	1.5 (0.9) out of 5
**My child moved less than usual during the study**	1.3 (0.7) out of 5
**I believe physical activity is a good way to measure how my child feels**	3.8 (1.0) out of 5
**The watch was comfortable**	3.8 (1.0) out of 5
**The watch hurt**	1.8 (1.1) out of 5
**My child like wearing the watch all day**	4.5 (0.8) out of 5
**I often forgot to make my child wear the watch**	1.2 (0.7) out of 5
**Measuring weight was tedious**	1.2 (0.7) out of 5
**Measuring blood pressure was tedious**	1.8 (1.2) out of 5
**Measuring temperature was tedious**	1.1(0.3) out of 5
**Measuring pulmonary function was tedious**	1.7 (1.2) out of 5
**I encountered technical problems during the study**	25% answered ‘Yes’
**Technical problems were due to**	‘Blood pressure monitor did not work.’
	‘Bluetooth connectivity issues.’
	‘Pulmonary function meter did not connect.’
