# Supplementary material for: Towards remote monitoring in pediatric care and clinical trials—Tolerability, repeatability and reference values of candidate digital endpoints derived from physical activity, heart rate and sleep in healthy children
Source: PLoS One. 2021 Jan 7;16(1):e0244877. doi: 10.1371/journal.pone.0244877 (PMC7790377; doi:10.1371/journal.pone.0244877)
Supplement: S2 Table — (PDF) [file pone.0244877.s007.pdf]

S2 Table. Model Coefficients of physical activity-related candidate endpoints.

Model 1. PA Daily			
**Predictors**	**Estimates**	**CI**	**p**
(Intercept)	8.21	-3.07 – 19.48	0.154
Age [1st degree]	-1.40	-9.85 – 7.04	0.744
Age [2nd degree]	24.06	8.34 – 39.77	**0.003**
Age [3rd degree]	-8.50	-14.35 – -2.65	**0.004**
Sex [Male]	-1.33	-10.41 – 7.75	0.775
Watch wear time (6:00-22:00)	0.69	0.60 – 0.77	**<0.001**
Rain duration (h)	-0.65	-0.86 – -0.45	**<0.001**
Temperature [1st degree]	8.89	4.75 – 13.03	**<0.001**
Temperature [2nd degree]	-3.27	-13.58 – 7.03	0.534
Temperature [3rd degree]	-8.05	-16.88 – 0.79	0.074
Weekday [Tue]	-1.36	-3.31 – 0.59	0.172
Weekday [Wed]	1.16	-0.80 – 3.13	0.247
Weekday [Thu]	0.70	-1.27 – 2.67	0.485
Weekday [Fri]	2.36	0.40 – 4.31	**0.018**
Weekday [Sat]	-0.05	-2.00 – 1.91	0.963
Weekday [Sun]	-8.20	-10.16 – -6.24	**<0.001**
Population density [> 2500 / km^2]	6.80	3.17 – 10.42	**<0.001**
Age [1st degree] * sex [Male]	7.86	-4.18 – 19.89	0.201
Age [2nd degree] * sex [Male]	15.93	-7.46 – 39.33	0.182
Age [3rd degree] * sex [Male]	-5.61	-15.60 – 4.39	0.271
**Random Effects**			
σ^2	248.12		
$\tau_{00\ SubjectNr}$	81.90		
ICC	0.25		
$N_{SubjectNr}$	174		
**Observations**	3529		
**Marginal R^2 / Conditional R^2**	0.287 / 0.464		

* Age and temperature were best described by a spline with 3 degrees of freedom. Estimates and confidence intervals are displayed on a square root scale.

Justification of inclusion of spline covariates in the final model

	**Linear relationship***	**2nd degree spline****	**3rd degree spline****	**4th degree spline****
**Age**	**ΔAIC +2, p = 0.45**	**ΔAIC -38, p < 0.001**	**ΔAIC -8, p = 0.002**	**ΔAIC +2, p = 0.39**
**Temperature**	**ΔAIC -3, p < 0.02**	**ΔAIC -2, p < 0.04**	**ΔAIC -8, p = 0.0015**	**ΔAIC -1, p = 0.08**

*** Compared to model without covariate**

**** Compared to variable in previous column**

Model 2. PA^{max}			
**Predictors**	**Estimates**	**CI**	**p**
(Intercept)	13.78	7.80 – 19.77	**<0.001**
Age [1st degree]	0.01	-3.78 – 3.81	0.994
Age [2nd degree]	11.66	4.57 – 18.74	**0.001**
Age [3rd degree]	-1.91	-4.55 – 0.72	0.154
Sex [Male]	-1.85	-5.95 – 2.26	0.377
Watch wear time (6:00-22:00)	0.21	0.16 – 0.26	**<0.001**
Rain duration (h)	-0.31	-0.43 – -0.20	**<0.001**
Temperature [1st degree]	3.55	1.30 – 5.80	**0.002**
Temperature [2nd degree]	-2.97	-8.82 – 2.87	0.318
Temperature [3rd degree]	-5.98	-11.05 – -0.90	**0.021**
Weekday [Sun]	-2.36	-3.24 – -1.49	**<0.001**
Population density [> 2500 / km^2]	2.98	1.33 – 4.63	**<0.001**
Age [1st degree] * sex [Male]	6.17	0.75 – 11.59	**0.026**
Age [2nd degree] * sex [Male]	7.58	-2.98 – 18.14	0.160
Age [3rd degree] * sex [Male]	-3.85	-8.35 – 0.64	0.093
**Random Effects**			
σ^2	84.79		
$\tau_{00\ SubjectNr}$	14.88		
ICC	0.15		
$N_{SubjectNr}$	174		
Observations	3529		
**Marginal R^2 / Conditional R^2**	0.178 / 0.301		

* Age and temperature were best described by a spline with 3 degrees of freedom. Estimates and confidence intervals are displayed on a square root scale

Model 3. Daily PA^{avg} (weekly)			
**Predictors**	**Estimates**	**CI**	**p**
(Intercept)	-4.45	-25.81 – 16.91	0.683
Age [1st degree]	-1.29	-9.69 – 7.12	0.764
Age [2nd degree]	23.51	7.94 – 39.08	**0.003**
Age [3rd degree]	-7.52	-13.32 – -1.71	**0.011**
Sex [Male]	-3.33	-12.26 – 5.59	0.464
Mean temperature [1st degree]	10.36	5.29 – 15.42	**<0.001**
Mean temperature [2nd degree]	2.57	-9.57 – 14.71	0.679
Mean temperature [3rd degree]	-0.28	-7.44 – 6.88	0.939
Mean rain duration (h)	-1.02	-1.59 – -0.44	**0.001**
Mean watch wear time (6:00-22:00)	0.79	0.58 – 1.00	**<0.001**
Population density [> 2500 / km^2]	6.21	2.53 – 9.90	**0.001**
Age [1st degree] * sex [Male]	9.24	-2.70 – 21.18	0.129
Age [2nd degree] * sex [Male]	20.40	-2.67 – 43.47	0.083
Age [3rd degree] * sex [Male]	-5.13	-15.04 – 4.77	0.310
**Random Effects**			
σ^2	54.17		
$\tau_{00\ SubjectNr}$	73.76		
ICC	0.58		
$N_{SubjectNr}$	174		
**Observations**	515		
**Marginal R^2 / Conditional R^2**	0.486 / 0.782		

* Age and temperature were best described by a spline with 3 degrees of freedom. Estimates and confidence intervals are displayed on a square root scale

Model 4. Daily PA^{90th} (weekly)			
**Predictors**	**Estimates**	**CI**	**p**
(Intercept)	7.00	-19.45 – 33.45	0.604
Age [1st degree]	4.81	-4.78 – 14.40	0.326
Age [2nd degree]	31.04	13.24 – 48.83	**0.001**
Age [3rd degree]	-4.03	-10.66 – 2.59	0.233
Sex [Male]	-4.19	-14.40 – 6.03	0.422
Mean temperature [1st degree]	12.78	6.48 – 19.09	**<0.001**
Mean temperature [2nd degree]	-0.11	-15.86 – 15.65	0.990
Mean temperature [3rd degree]	-3.77	-13.01 – 5.46	0.423
Mean rain duration (h)	-0.98	-1.72 – -0.24	**0.009**
Mean watch wear time (6:00-22:00)	0.78	0.52 – 1.04	**<0.001**
Population density [> 2500 / km^2]	6.93	2.70 – 11.17	**0.001**
Age [1st degree] * sex [Male]	11.13	-2.51 – 24.77	0.110
Age [2nd degree] * sex [Male]	24.18	-2.18 – 50.53	0.072
Age [3rd degree] * sex [Male]	-7.22	-18.51 – 4.08	0.210
**Random Effects**			
σ^2	97.40		
$\tau_{00\ SubjectNr}$	86.74		
ICC	0.47		
$N_{SubjectNr}$	174		
**Observations**	515		
**Marginal R^2 / Conditional R^2**	0.461 / 0.715		

* Age and temperature were best described by a spline with 3 degrees of freedom. Estimates and confidence intervals are displayed on a square root scale

Model 5. Daily PA^{10th}			
**Predictors**	**Estimates**	**CI**	**p**
(Intercept)	-37.87	-60.63 – -15.11	**0.001**
Age [1st degree]	-6.63	-13.68 – 0.43	0.066
Age [2nd degree]	23.32	10.48 – 36.16	**<0.001**
Age [3rd degree]	-14.03	-19.24 – -8.82	**<0.001**
sex [Male]	4.62	1.36 – 7.88	**0.005**
Mean temperature	0.38	0.09 – 0.68	**0.011**
Mean rain duration (h)	-0.97	-1.60 – -0.35	**0.002**
Mean watch wear time (6:00-22:00)	0.97	0.74 – 1.20	**<0.001**
Urbanisation [Extremely urbanized]	5.70	1.75 – 9.65	**0.005**
**Random Effects**			
σ^2	67.49		
$\tau_{00\ SubjectNr}$	90.23		
ICC	0.57		
$N_{SubjectNr}$	174		
**Observations**	515		
**Marginal R^2 / Conditional R^2**	0.398 / 0.742		

* Age was described best with a spine with 3 degrees of freedom. Estimates are not transformed

Model 6. Hourly PA^{90th} (weekly)			
**Predictors**	**Estimates**	**CI**	**p**
(Intercept)	12.39	2.24 – 22.55	**0.017**
Age [1st degree]	5.19	0.04 – 10.35	**0.048**
Age [2nd degree]	-2.74	-5.13 – -0.36	**0.024**
sex [Male]	-2.25	-5.40 – 0.89	0.160
Mean rain duration (h)	-0.46	-0.71 – -0.21	**<0.001**
Mean temperature [1st degree]	4.44	2.30 – 6.58	**<0.001**
Mean temperature [2nd degree]	0.53	-4.76 – 5.83	0.844
Mean temperature [3rd degree]	-0.83	-3.78 – 2.12	0.582
Population density [> 2500 / km^2]	-2.10	-3.59 – -0.62	**0.005**
Mean watch wear time (6:00-22:00)	0.17	0.07 – 0.27	**0.001**
Age [1st degree] * sex [Male]	12.40	5.26 – 19.55	**0.001**
Age [2nd degree] * sex [Male]	-3.97	-7.93 – -0.00	**0.050**
**Random Effects**			
σ^2	10.74		
$\tau_{00\ SubjectNr1}$	11.82		
ICC	0.52		
N $_{SubjectNr1}$	174		
**Observations**	515		
**Marginal R^2 / Conditional R^2**	0.448 / 0.737		

* Age and temperature were best described by a spline with 2 and 3 degrees of freedom, respectively. Estimates and confidence intervals are displayed on a square root scale.

Model 7. Hourly PA^{50th}			
**Predictors**	**Estimates**	**CI**	**p**
(Intercept)	3.22	2.36 – 4.07	**<0.001**
Age [1st degree]	-0.07	-0.30 – 0.15	0.519
Age [2nd degree]	0.73	0.30 – 1.16	**0.001**
Age [3rd degree]	-0.44	-0.62 – -0.27	**<0.001**
sex [Male]	0.14	0.03 – 0.25	**0.016**
Mean watch wear time (6:00-22:00)	0.02	0.01 – 0.03	**<0.001**
**Random Effects**			
σ^2	0.07		
$\tau_{00\ SubjectNr1}$	0.11		
ICC	0.60		
N $_{SubjectNr1}$	174		
**Observations**	515		
**Marginal R^2 / Conditional R^2**	0.228 / 0.693		

* Age was best described by a spline with 3 degrees of freedom. Estimates and confidence intervals are displayed on a log scale.
