# Supplementary material for: Towards remote monitoring in pediatric care and clinical trials—Tolerability, repeatability and reference values of candidate digital endpoints derived from physical activity, heart rate and sleep in healthy children
Source: PLoS One. 2021 Jan 7;16(1):e0244877. doi: 10.1371/journal.pone.0244877 (PMC7790377; doi:10.1371/journal.pone.0244877)
Supplement: S3 Table — (PDF) [file pone.0244877.s008.pdf]

S3 Table. Model coefficients of heart rate parameters

Model 1. Daytime heart rate (bpm)			
**Predictors**	**Estimates**	**CI**	**p**
**(Intercept)**	110.04	107.35 – 112.72	<0.001
**Age [1st degree]**	-14.63	-18.09 – -11.17	<0.001
**Age [2nd degree]**	-54.69	-61.28 – -48.10	<0.001
**Age [3rd degree]**	-21.23	-24.03 – -18.43	<0.001
**sex [Male]**	-2.71	-4.43 – -0.99	0.002
**Random Effects**			
σ^2	21.94		
$\tau_{00\ SubjectNr}$	30.83		
ICC	0.58		
N $_{SubjectNr}$	170		
**Observations**	3244		
**Marginal R^2 / Conditional R^2**	0.611 / 0.838		

* Age was best described by a spline with 3 degrees of freedom. Estimates are not transformed.

Justification of inclusion of spline covariates in the final model for daytime heart rate

	**Linear relationship***	**2nd degree spline****	**3rd degree spline****	**4th degree spline****
Age	ΔAIC -191, $p < 0.001$	ΔAIC -11, $p < 0.001$	ΔAIC -25, $p < 0.001$	ΔAIC +2, $p = 0.71$

* Compared to model without covariate ** Compared to variable in previous column

Model 2. Nocturnal heart rate (bpm)			
**Predictors**	**Estimates**	**CI**	**p**
**(Intercept)**	94.78	91.70 – 97.87	**<0.001**
**Age [1st degree]**	-13.05	-17.20 – -8.90	<0.001
**Age [2nd degree]**	-54.59	-62.48 – -46.70	<0.001
**Age [3rd degree]**	-18.03	-21.34 – -14.72	<0.001
**Random Effects**			
**σ^2**	31.31		
**$\tau_{00\ SubjectNr}$**	44.02		
**ICC**	0.58		
**N $_{SubjectNr}$**	172		
**Observations**	3404		
**Marginal R^2 / Conditional R^2**	0.497 / 0.791		

* Estimates are not transformed.

Justification of inclusion of spline covariates in the final model for nocturnal heart rate

	**Linear relationship***	**2nd degree spline****	**3rd degree spline****	**4th degree spline****
Age	ΔAIC -132, $p < 0.001$	ΔAIC -14, $p < 0.001$	ΔAIC -21, $p < 0.001$	ΔAIC +2, $p = 1$

* Compared to model without covariate ** Compared to variable in previous column

**Model 3. Correlation heart rate and step count**			
**Predictors**	Estimates	CI	p
**(Intercept)**	106.22	103.29 – 109.15	<0.001
**stepsTotalDaily1000**	-0.14	-0.39 – 0.11	0.272
**Age**	-2.53	-2.82 – -2.24	<0.001
**stepsTotalDaily1000 * Age**	0.08	0.05 – 0.10	<0.001
**Random Effects**			
**σ2**	18.59		
**τ00 SubjectNr**	59.85		
**τ11 SubjectNr.stepsTotalDaily1000**	0.24		
**ρ01 SubjectNr**	-0.54		
**ICC**	0.71		
**$N_{SubjectNr}$**	170		
**Observations**	3244		
**Marginal R^2 / Conditional R^2**	0.556 / 0.869		

* Estimates are not transformed.
