# Supplementary material for: Towards remote monitoring in pediatric care and clinical trials—Tolerability, repeatability and reference values of candidate digital endpoints derived from physical activity, heart rate and sleep in healthy children
Source: PLoS One. 2021 Jan 7;16(1):e0244877. doi: 10.1371/journal.pone.0244877 (PMC7790377; doi:10.1371/journal.pone.0244877)
Supplement: S4 Table — (PDF) [file pone.0244877.s009.pdf]

S4 Table. Model coefficients of accelerometer-derived sleep parameters

Model 1. Sleep duration (hours)			
**Predictors**	**Estimates**	**CI**	**p**
**(Intercept)**	9.56	9.30 – 9.82	**<0.001**
**Age [1st degree]**	-0.80	-1.36 – -0.24	**0.005**
**Age [2nd degree]**	-1.35	-1.64 – -1.05	**<0.001**
**sex [Male]**	-0.29	-0.48 – -0.11	**0.002**
**Random Effects**			
**σ^2**	1.14		
**τ_{00} SubjectNr**	0.32		
**ICC**	0.22		
**N SubjectNr**	173		
**Observations**	3115		
**Marginal R^2 / Conditional R^2**	0.116 / 0.308		

* Age was best described by a spline with 2 degrees of freedom. Estimates are not transformed.

Justification of inclusion of spline covariate in the final model

	**Linear relationship***	**2nd degree spline****	**3rd degree spline****
Age	ΔAIC -65, $p < 0.001$	ΔAIC -10, $p < 0.001$	ΔAIC -1, $p = 0.09$

* Compared to model without covariate ** Compared to variable in previous column

Model 2. Sleep depth (% light sleep)			
**Predictors**	**Estimates**	**CI**	**p**
**(Intercept)**	52.73	50.37 – 55.10	**<0.001**
**Age**	0.29	0.08 – 0.51	**0.006**
**sex [Male]**	2.47	0.66 – 4.27	**0.008**
**Random Effects**			
**σ2**	42.43		
**τ00 SubjectNr**	33.35		
**ICC**	0.44		
**N SubjectNr**	172		
**Observations**	3020		
**Marginal R2 / Conditional R2**	0.034 / 0.459		

* Estimates are not transformed.

Model 3. Wakeup count (n)			
**Predictors**	**Incidence Rate Ratios**	**CI**	**p**
**(Intercept)**	2.90	2.29 – 3.68	<0.001
**Age**	0.95	0.92 – 0.97	<0.001
**Random Effects**			
**σ2**	0.48		
**τ00 SubjectNr**	0.41		
**ICC**	0.46		
**N SubjectNr**	172		
**Observations**	3020		
**Marginal R2 / Conditional R2**	0.057 / 0.488		

* Model was fitted assuming a negative binomial distribution with logarithmic link function.
