# Supplementary material for: Towards remote monitoring in pediatric care and clinical trials—Tolerability, repeatability and reference values of candidate digital endpoints derived from physical activity, heart rate and sleep in healthy children
Source: PLoS One. 2021 Jan 7;16(1):e0244877. doi: 10.1371/journal.pone.0244877 (PMC7790377; doi:10.1371/journal.pone.0244877)
Supplement: S5 Table — (PDF) [file pone.0244877.s010.pdf]

S5 Table. Dataset legend and explanation

Parameter	Legend	Additional information
**SubjectNr**	Subject number, unique for each participant	Anonymized.
**DayNo**	Study day number,	Day 0 (screening day, incomplete data expected)<br>Day 1-21 (study days)
**weekday**	Day of the week	
**dayType**	Type of day (school, weekend, holiday)	
**Age**	Age of participant	
**sex**	Sex of participant	
**weight**	Weight of participant	NA: data not provided by participant.
**height**	Height of participant	NA: data not provided by participant.
**BMI_SDS**	Standard deviation score of participant's BMI (based on Dutch reference values)	NA: BMI unknown
**ethnicity**	Ethnicity of participant	
**school_year_final**	School type of participant	
**sportsyesno**	Whether subject regularly plays sports or not.	
**urbanisation**	Urbanization grade of city of residence of subject:<br>- Extremely urbanized: > 2500 households / km^2<br>- Very urbanized: 1500-2500 households / km^2<br>- Moderately urbanized: 1000-1500 households / km^2<br>- Little urbanized: 500-1000 households / km^2	
**PedsQL_score_baseline**	PedsQL 4.0 score	Completed at day 0 (screening day).<br>NA: subject did not complete questionnaire.
**stepsTotalDaily**	Total step count taken during that day	NA or 0: No data available. Missing data either due to noncompliance or data-connectivity issue.

**steps_hour_max**	Steps taken during the most active hour of each day.	
**steps00**	Step count between 00:00AM and 01:00AM	NA or 0: No data available. Missing data either due to noncompliance, sleep/non-movement, or data-connectivity issue.
**steps01**	Step count between 01:00AM and 02:00AM	"
**steps02**	Step count between 02:00AM and 03:00AM	"
**steps03**	Step count between 03:00AM and 04:00AM	"
**steps04**	Step count between 04:00AM and 05:00AM	"
**steps05**	Step count between 05:00AM and 06:00AM	"
**steps06**	Step count between 06:00AM and 07:00AM	"
**steps07**	Step count between 07:00AM and 08:00AM	"
**steps08**	Step count between 08:00AM and 09:00AM	"
**steps09**	Step count between 09:00AM and 10:00AM	"
**steps10**	Step count between 10:00AM and 11:00AM	"
**steps11**	Step count between 11:00PM and 12:00PM	"
**steps12**	Step count between 12:00PM and 01:00PM	"
**steps13**	Step count between 01:00PM and 02:00PM	"
**steps14**	Step count between 02:00PM and 03:00PM	"
**steps15**	Step count between 03:00PM and 04:00PM	"
**steps16**	Step count between 04:00PM and 05:00PM	"
**steps17**	Step count between 05:00PM and 06:00PM	"
**steps18**	Step count between 06:00PM and 07:00PM	"
**steps19**	Step count between 07:00PM and 08:00PM	"
**steps20**	Step count between 08:00PM and 09:00PM	"

**steps21**	Step count between 09:00PM and 10:00PM	“
**steps22**	Step count between 10:00PM and 11:00PM	“
**steps23**	Step count between 11:00PM and 12:00AM	“
**HR05Perc**	5th percentile of all heart rates measured during a day.	NA: not enough heart rate data available during this day to calculate 5th percentile.
**HR95Perc**	95th percentile of all heart rates measured during a day.	NA: not enough heart rate data available during this day to calculate 95th percentile.
**HRMinSleep**	Minimum heart rate measured during sleep	NA: no heart rate registered during sleep.
**HRMaxSleep**	Maximum heart rate measured during sleep	NA: no heart rate registered during sleep.
**AVGHR_daily**	Average heart rate during a day	NA: not enough hourly heart rate data available during this day to calculate average
**AVGHR_sleep**	Average heart rate during 12:00AM and 05:00AM	NA: not enough hourly heart rate data available during this period to calculate average
**AVGHR_wake**	Average heart rate during 6:00AM and 22:00PM	NA: not enough hourly heart rate data available during this period to calculate average
**HR00**	Heart rate between 00:00AM and 01:00AM	NA: no heart rate reglistered during this hour. Either due to noncompliance, inadequate device handling or data connectivity-issue.
**HR01**	Heart rate between 01:00AM and 02:00AM	“
**HR02**	Heart rate between 02:00AM and 03:00AM	“
**HR03**	Heart rate between 03:00AM and 04:00AM	“
**HR04**	Heart rate between 04:00AM and 05:00AM	“
**HR05**	Heart rate between 05:00AM and 06:00AM	“
**HR06**	Heart rate between 06:00AM and 07:00AM	“

**HR07**	Heart rate between 07:00AM and 08:00AM	"
**HR08**	Heart rate between 08:00AM and 09:00AM	"
**HR09**	Heart rate between 09:00AM and 10:00AM	"
**HR10**	Heart rate between 10:00AM and 11:00AM	"
**HR11**	Heart rate between 11:00PM and 12:00PM	"
**HR12**	Heart rate between 12:00PM and 01:00PM	"
**HR13**	Heart rate between 01:00PM and 02:00PM	"
**HR14**	Heart rate between 02:00PM and 03:00PM	"
**HR15**	Heart rate between 03:00PM and 04:00PM	"
**HR16**	Heart rate between 04:00PM and 05:00PM	"
**HR17**	Heart rate between 05:00PM and 06:00PM	"
**HR18**	Heart rate between 06:00PM and 07:00PM	"
**HR19**	Heart rate between 07:00PM and 08:00PM	"
**HR20**	Heart rate between 08:00PM and 09:00PM	"
**HR21**	Heart rate between 09:00PM and 10:00PM	"
**HR22**	Heart rate between 10:00PM and 11:00PM	"
**HR23**	Heart rate between 11:00PM and 12:00AM	"
**Wear05H**	Wear time (percentage) of the smartwatch between 00AM and 05:00 AM	Wear time was calculated by appraising both HR and step count data during each hour. If either was present, the hour was classified as 'worn'
**wear16H**	Wear time (percentage) of the smartwatch between 6:00AM and 10:00PM	Wear time was calculated by appraising both HR and step count data during each hour. If either was present, the hour was classified as 'worn'

**wear24H**	Wear time (percentage) of the smartwatch during a day	Wear time was calculated by appraising both HR and step count data during each hour. If either was present, the hour was classified as 'worn'
**BODY_TEMPERATURE_DEG_C**	Body temperature (degrees Celsius)	NA: measurement was not performed on this day.
**DIASTOLIC_BLOOD_PRESSURE_MMHG**	Diastolic blood pressure (mmHg)	NA: measurement was not performed on this day.
**HEART_PULSE_BPM**	Heart rate (bpm) measured by blood pressure monitor	NA: measurement was not performed on this day.
**SYSTOLIC_BLOOD_PRESSURE_MMHG**	Systolic blood pressure (mmHg)	NA: measurement was not performed on this day.
**WEIGHT_KG**	Weight measured by scales (kg)	NA: measurement was not performed on this day.
**awakeDuration**	Time in seconds the subject was awake	NA: no sleep data was registered. Most likely due to not wearing the watch. Other causes: non-detection of sleep by the algorithm, data-connectivity issue.
**lightSleepDuration**	Time in seconds the subject slept with light depth	NA: no sleep data was registered. Most likely due to not wearing the watch. Other causes: non-detection of sleep by the algorithm, data-connectivity issue.
**deepSleepDuration**	Time in seconds the subject slept with deep depth	NA: no sleep data was registered. Most likely due to not wearing the watch. Other causes: non-detection of sleep by the algorithm, data-connectivity issue.
**wakeUpCount**	Number of times the subject woke up	NA: no sleep data was registered. Most likely due to not wearing the watch. Other causes: non-detection of sleep by the algorithm, data-connectivity issue.
**sleeptime**	Clock time the subject started sleeping	NA: no sleep data was registered. Most likely due to not wearing the watch. Other causes: non-detection of sleep by the algorithm, data-connectivity issue.

**waketime**	Clock time the subject woke up	NA: no sleep data was registered. Most likely due to not wearing the watch. Other causes: non-detection of sleep by the algorithm, data-connectivity issue.
**fvc_best**	Forced vital capacity (liters)	NA: measurement was not performed on this day.
**fev1_best**	Forced expiratory volume (liters)	NA: measurement was not performed on this day.
**pef_best**	Peak flow (liters per second)	NA: measurement was not performed on this day.
**grade_fev1**	Spirometry maneuver quality graded by physician for FEV1	Grading according to ATS criteria.<br>NA: measurement was not performed on this day.
**grade_fvc**	Spirometry maneuver quality graded by physician for FVC	Grading according to ATS criteria.<br>NA: measurement was not performed on this day.
**predicted_fvc_best**	Predicted FVC for this subject	NA: measurement was not performed on this day.
**predicted_fev1_best**	Predicted FEV1 for this subject	NA: measurement was not performed on this day.
**predicted_fev1_ratio_best**	Predicted FEV1/FVC ratio for this subject	NA: measurement was not performed on this day.
**fev1_ratio_best**	FEV1/FVC ratio	NA: measurement was not performed on this day.
**fev1_percentage**	Percentage of predicted FEV1	NA: measurement was not performed on this day.
**fvc1_percentage**	Percentage of predicted FVC	NA: measurement was not performed on this day.
**fev1_ratio_percentage**	Percentage of predicted FEV1/FVC ratio	NA: measurement was not performed on this day.
**school_yes_no**	Questionnaire data regarding whether subject went to school, daycare or neither.	NA: questionnaire was not completed on this day.
**screentime**	Questionnaire data regarding the duration of screentime (phone, computer, tablet, tv) during the day.	NA: questionnaire was not completed on this day.
**FG**	Mean wind speed (m/s)	
**FHX**	Highest hourly mean wind speed (m/s)	

**FHN**	Lowest hourly mean wind speed (m/s)	
**TG**	Mean temperature (degrees Celsius)	
**TN**	Minimum temperature (degrees Celsius)	
**TX**	Maximum temperature (degrees Celsius)	
**SQ**	Sunshine duration (hours)	
**SP**	Sunshine duration (percentage of maximum possible duration)	
**DR**	Rain duration (hours)	
**RH**	Rainfall (0.1 mm)	
**RHX**	Highest hourly rainfall (0.1 mm)	
