# Supplementary material for: Towards remote monitoring in pediatric care and clinical trials—Tolerability, repeatability and reference values of candidate digital endpoints derived from physical activity, heart rate and sleep in healthy children
Source: PLoS One. 2021 Jan 7;16(1):e0244877. doi: 10.1371/journal.pone.0244877 (PMC7790377; doi:10.1371/journal.pone.0244877)
Supplement: S2 File — (PDF) [file pone.0244877.s013.pdf]

Vragenlijst einde studieperiode

Nederlands - Ouders

Ziekte en bijwerkingen

Is uw kind tijdens het onderzoek ziek geweest? (ja/nee)

Zo ja, kunt u dit omschrijven? (tekst)

Is er tijdens het onderzoek iets gebeurd wat u als bijwerking van de studieapparaten ziet? (ja/nee)

Zo ja, hoe erg was dit? ___

Zo ja, hoe lang duurde dit? ___

Impact

Mijn kind viel meer op voor leeftijdsgenoten tijdens het gebruik van het horloge. (1-5)

Mijn kind werd op school door leraren aangesproken op het gebruik van het horloge. (1-5)

Mijn kind heeft op school nadelen, zoals plagen of pesten, ondervonden door het dragen van het horloge. (1-5)

Mijn kind vond het leuk om het horloge te dragen. (1-5)

Mijn kind is blij dat de onderzoeksperiode voorbij is. (1-5)

Mijn kind heeft zich anders gedragen dan normaal tijdens het dragen van het horloge. (1-5)

Mijn kind heeft meer bewogen dan normaal tijdens het dragen van het horloge. (1-5)

Mijn kind heeft minder bewogen dan normaal tijdens het dragen van het horloge. (1-5)

Mijn kind heeft thuis vaak over het onderzoek gepraat. (1-5)

Comfort

Hoe zou u het comfort van het horloge beoordelen? (1-5)

Heeft het horloge pijn gedaan? (1-5)

Zo ja, hoe vaak heeft zich dit voorgedaan (tekst)

Heeft u zorgen over de veiligheid van de studie apparaten? (ja / nee)

Zo ja, kunt u dit uitleggen? (tekst)

De manier van het meten van het gewicht vond ik:

Vervelend 1-2-3-4-5 aangenaam
Moeilijk 1-2-3-4-5 makkelijk
Niet nuttig 1-2-3-4-5 nuttig
Onbetrouwbaar 1-2-3-4-5 betrouwbaar

De manier van het meten van de bloeddruk vond ik

Vervelend 1-2-3-4-5 aangenaam
Moeilijk 1-2-3-4-5 makkelijk Niet nuttig
1-2-3-4-5 nuttig Onbetrouwbaar 1-2-3-
4-5 betrouwbaar

De manier van het meten van de longfunctie vond ik:

Vervelend 1-2-3-4-5 aangenaam

Moeilijk 1-2-3-4-5 makkelijk Niet nuttig 1-2-3-4-5 nuttig Onbetrouwbaar 1-2-3-4-5 betrouwbaar

De manier van het meten van de temperatuur vond ik:

Vervelend 1-2-3-4-5 aangenaam

Moeilijk 1-2-3-4-5 makkelijk Niet nuttig 1-2-3-4-5 nuttig Onbetrouwbaar 1-2-3-4-5 betrouwbaar

Belasting

Hoeveel tijd waren u en uw kind gemiddeld per dag kwijt aan de onderzoekstaken? (minuten)

Ik vond de hoeveelheid tijd die ik nodig had:

minimaal – weinig – voldoende – veel – te veel

Was de totale duur van de studie acceptabel voor u en uw kind?

ja - nee

Zo nee, hoeveel dagen zou voor u acceptabel zijn? _______

Heeft u het gevoel dat uw kind op alle dagen het horloge heeft gedragen?

Ja - nee

Zo nee, op hoeveel dagen is dit niet gelukt? _______

Hoe vond uw kind het om het horloge de hele dag aan te houden?

Heel moeilijk 1-2-3-4-5 helemaal niet moeilijk

Bent u tijdens de studie vergeten het horloge om te doen?

Weinig 1-2-3-4-5 vaak

Was het moeilijk om uw kind te overtuigen de apparaten te gebruiken?

Niet moeilijk 1-2-3-4-5 erg moeilijk

Ik vond het gebruik van de Nokia HealthMate app

makkelijk 1-2-3-4-5 moeilijk

Ik vond het gebruik van de longfunctie app

Makkelijk 1-2-3-4-5 moeilijk

Ik vond het invullen van de digitale vragenlijsten

Makkelijk 1-2-3-4-5 moeilijk

Ik heb tijdens het onderzoek veel technische problemen ondervonden. (ja/nee)

Zo ja, wat voor problemen waren dit? (tekst)

Hoe frequent traden deze problemen op? ____

Heeft u ideeën om eventuele vervolgonderzoeken beter te laten verlopen? (tekst)

Ik geloof dat het meten van de lichamelijk activiteit een goede inschatting geeft over hoe mijn kind zich voelt.

Oneens 1-2-3-4-5 Eens

Zouden u en uw kind in de toekomst met vergelijkbare onderzoeken mee doen? (ja/nee)

Nederlands - Kinderen (> 12 jaar oud)

Adverse events

Ben je tijdens het onderzoek ziek geweest? (ja/nee)

Zo ja, kun je dit omschrijven? (tekst)

Is er tijdens het onderzoek iets gebeurd wat je als bijwerking van de studieapparaten ziet? (ja/nee)

Zo ja, hoe erg was dit? ___

Zo ja, hoe lang duurde dit? ___

Impact

Ik viel meer op voor leeftijdsgenoten tijdens het gebruik van het horloge. (1-5)

Ik werd op school door leraren aangesproken op het gebruik van het horloge. (1-5)

Ik ben op school geplaagd of gepest door het dragen van het horloge. (1-5)

Ik vond het leuk om het horloge te dragen. (1-5)

Ik ben blij dat de onderzoeksperiode voorbij is. (1-5)

Ik heb me anders gedragen dan normaal tijdens het dragen van het horloge. (1-5)

Ik heb meer bewogen dan normaal tijdens het dragen van het horloge. (1-5)

Ik heb minder bewogen dan normaal tijdens het dragen van het horloge. (1-5)

Ik heb thuis vaak over het onderzoek gepraat. (1-5)

Comfort

Hoe lekker vond je het horloge zitten? (1-5)

Heeft het horloge pijn gedaan? (1-5)

Zo ja, hoe vaak heeft het pijn gedaan? (tekst)

De manier van het meten van het gewicht vond ik:

Vervelend 1-2-3-4-5 aangenaam

Moeilijk 1-2-3-4-5 makkelijk

De manier van het meten van de bloeddruk vond ik

Vervelend 1-2-3-4-5 aangenaam

Moeilijk 1-2-3-4-5 makkelijk

De manier van het meten van de longfunctie vond ik:

Vervelend 1-2-3-4-5 aangenaam

Moeilijk 1-2-3-4-5 makkelijk

De manier van het meten van de temperatuur vond ik:

Vervelend 1-2-3-4-5 aangenaam

Moeilijk 1-2-3-4-5 makkelijk

Belasting

Hoeveel tijd je gemiddeld per dag kwijt aan het onderzoek? (minuten)

Ik vond de hoeveelheid tijd die ik nodig had:

minimaal – weinig – voldoende – veel – te veel

Vond je het onderzoek kort of lang duren? (lang/kort/geen van beiden)

Heb je elke dag het horloge gedragen?

Ja - nee

Zo nee, op hoeveel dagen is dit niet gelukt? _______

Hoe vond je het om het horloge de hele dag aan te houden?

Heel moeilijk 1-2-3-4-5 helemaal niet moeilijk

Bent je tijdens de studie vergeten het horloge om te doen?

Weinig 1-2-3-4-5 vaak

Ik vond het gebruik van de Nokia HealthMate app

makkelijk 1-2-3-4-5 moeilijk

Ik vond het gebruik van de longfunctie app

Makkelijk 1-2-3-4-5 moeilijk

Ik vond het invullen van de digitale vragenlijsten

Makkelijk 1-2-3-4-5 moeilijk

Ik heb tijdens het onderzoek veel technische problemen ondervonden. (ja/nee)

Zo ja, wat voor problemen waren dit? (tekst)

Hoe vaak kwam dit voor? ____

Heeft je ideeën om eventuele vervolgonderzoeken beter te laten verlopen? (tekst)

Zou je in de toekomst met dit soort onderzoek mee doen? (ja/nee)

Nederlands - Kinderen (> 6 jaar en < 12 jaar oud, via interview)

Vond je het leuk om het horloge te dragen? (ja/nee)

Zo nee, waarom niet?

Heb je het horloge afgedaan, omdat het pijn deed? (nee/soms/vaak)

Heb je het horloge afgedaan, omdat je er geen zin in had? (nee/soms/vaak)

Ben jij blij, of vind je het jammer dat je het horloge niet meer om hoeft. (blij/jammer/maakt niet uit)

Vond je het onderzoek kort of lang duren? (lang/kort/geen van beiden)

Vond je de nieuwe thermometer fijner, of minder fijn dan de thermometer in het ziekenhuis? (fijner/minder fijn/maakt niet uit)

Vond je het leuk of niet leuk om elke dag je longen te testen. (leuk/niet leuk/maakt niet uit)

Zo nee, waarom niet?

Hoe vond je het om zelf in te schatten hoe veel je hebt bewogen op een dag? (helemaal niet moeilijk/ redelijk moeilijk/moeilijk/heel moeilijk

End-of-study-questionnaire

English - Parents

Adverse events

Has your child been sick during this study? (yes/no)

If so, could you elaborate? (tekst)

Has something occurred during the study period that you consider as side effect of the study devices? (yes/no)

If so, how bad was it? ___

If so, how long did it take to resolve? ___

Impact

My child was noticed more by his peers while wearing the watch. (1-5)

My child was approached by a teacher about wearing the watch. (1-5)

My child has experienced negative remarks, was teased or bullied because he/she was wearing the watch. (1-5)

My child liked wearing the watch(1-5)

My child is happy the study trial is over. (1-5)

My child acted different, when he/she wore the watch. (1-5)

My child was more active, when he/she wore the watch.(1-5)

My child was less active, when he/she wore the watch (1-5)

My child frequently talked about the research at home. (1-5)

Comfort

How would you rate the comfort of the watch? (1-5)

Did the watch hurt? (yes/no)

If yes, how frequent did it hurt?(text)

Did you have any concern about the safety of the devices? (0-1)

If so, could you elaborate? (text)

The method of measuring lung function was:

Uncomfortable 1-2-3-4-5 comfortable
Hard 1-2-3-4-5 Easy Useless 1-2-3-
4-5 useful Untrustworthy 1-2-3-4-5
trustworthy

The method of measuring the temperature was

Uncomfortable 1-2-3-4-5 comfortable
Hard 1-2-3-4-5 Easy
Useless 1-2-3-4-5 useful
Untrustworthy 1-2-3-4-5 trustworthy

Burden

How much time did you and your child approximately spend on this study each day? (minutes) I regarded this amount of time spent on the study

Minimal – low – average – high – too much

Was the study duration acceptable for you and your child? (yes/no)

If no, what would be an acceptable amount of days? _____days

Do you think that your child wore the watch continuously? (yes/no)

If no, how many days didn't your child wear the watch? ______days

How hard was it for your child to wear the watch continuously?

Not hard 1-2-3-4-5 very hard

Did you forget to let your child wear the watch?

Sometimes 1-2-3-4-5 often

Was it hard to convince your child to use the devices?

Not hard 1-2-3-4-5 very hard

I found the use of the HealthMate application was:

Easy 1-2-3-4-5 hard

The lung function application was

Easy 1-2-3-4-5 hard

I found the completion of digital questionnaires

Easy 1-2-3-4-5 hard

Were there technical problems during the trial? (yes/no)

If yes, what were the observed problems? (text)

How frequent were the technical problems? ______ times

Do you think physical activity is a good estimate of the wellbeing of your child?

Disagree 1-2-3-4-5 agree

Would you and your child participate in this kind of research in the future? (yes/no)

Do you have any feedback for us to improve future studies? (text)

English - Children (> 12 year, via interview)

Adverse events

Have you been sick during this study? (yes/no)

If so, could you elaborate? (tekst)

Has something occurred during the study period that you consider as side effect of the study devices? (yes/no)

If so, how bad was it? ___

If so, how long did it take to resolve? ___

Impact

I was noticed more by other children while wearing the watch. (1-5)

I was approached by a teacher about wearing the watch. (1-5)

I have been teased or bullied because I was wearing the watch. (1-5)

I liked wearing the watch(1-5)

I am happy the study trial is over. (1-5)

I acted different when I wore the watch. (1-5)

I was more active, when I wore the watch. (1-5)

I was less active, when I wore the watch (1-5)

I frequently talked about the research at home. (1-5)

Comfort

Did the watch feel comfortable? (1-5)

Did the watch hurt? (yes/no)

If yes, how frequent did it hurt?(text)

The method of measuring lung function was:

Uncomfortable 1-2-3-4-5 comfortable

Hard 1-2-3-4-5 Easy

The method of measuring weight was

Uncomfortable 1-2-3-4-5 comfortable

Hard 1-2-3-4-5 Easy

The method of measuring blood pressure was

Uncomfortable 1-2-3-4-5 comfortable

Hard 1-2-3-4-5 Easy

Burden

How much time did you approximately spend on this study each day? (minutes)

I regarded this amount of time spent on the study:

Minimal – low – average – high – too much

Did you find the duration of this study to be long or short? (long/short/ just fine)

Do you wear the watch continuously? (yes/no)

If no, how many days didn't you child wear the watch? _____days

How hard was it for you to wear the watch continuously?

Not hard 1-2-3-4-5 very hard

Did you forget to wear the watch?

Sometimes 1-2-3-4-5 often

I found the use of the HealthMate application was:

Easy 1-2-3-4-5 hard

The lung function application was

Easy 1-2-3-4-5 hard

I found the completion of digital questionnaires

Easy 1-2-3-4-5 hard

Were there technical problems during the trial? (yes/no)

If yes, what were the problems? (text)

How frequent were the technical problems? ______ times

Would you participate in this kind of research in the future? (yes/no)

Do you have any feedback for us to improve future studies? (text)

English - Children (> 6 year and < 12 year, via interview)

Did you like to wear the watch?(yes/no)

If no, why not?

Did you remove the watch, because it hurt? (never – sometimes - often)

Did you remove the watch, because you did not feel like wearing it? (never – sometimes - often)

Are you happy or sad that you don’t have to wear the watch anymore. (happy/sad/don’t care)

Did you find the duration of this study to be long or short? (long/short/ just fine)

Did you like to test your lungs?(yes/no/don’t care)

Did you like to measure your blood pressure? (yes/no/ don’t care)

Did you like to measure your weight? (yes/no/ don’t care)
