# Supplementary material for: Towards remote monitoring in pediatric care and clinical trials—Tolerability, repeatability and reference values of candidate digital endpoints derived from physical activity, heart rate and sleep in healthy children
Source: PLoS One. 2021 Jan 7;16(1):e0244877. doi: 10.1371/journal.pone.0244877 (PMC7790377; doi:10.1371/journal.pone.0244877)
Supplement: Attachment — Submitted filename: Review_ PLOS ONE D 20-30878.pdf [file pone.0244877.s014.pdf]

This study examines the compliance and tolerability of using wearable and portable devices for home monitoring in healthy children between 2 and 16 years old. In addition, the study aims to determine reference values of candidate digital endpoints derived from physical activity, hear rate and sleep data in this population. This work presents an effort towards remote monitoring in pediatric population and holds the potential to provide useful guide for future clinical trials. My major comments are provided below.

1. In the Analysis Section (page 5, line 115), the calculation of the tolerability was discussed. However, little description was provided regarding how the repeatability was quantified in the paper.

2. In the Results Section, please provide test statistic, p value, and effect size for the findings in the study. For example, when reporting rain duration (Figure 1C) and ambient temperature (Figure 1D) were significantly associated with Daily PA (page 9, line 206), no test statistics were reported to support the conclusion. Similarly, when reporting sleep parameters (lines 253-262), test statistic, p value, effect size and confidence interval should be reported in a consistent way throughout the paper. When reporting a statistically significant correlation between HR and step count (line 248), test statistic and p value should be provided in the text to support the conclusion.

3. Table 3 includes 7 candidate endpoints, each of which was examined with respect to multiple factors (e.g., age, sex, wear time). The large number of tests conducted could lead to inflated false positives. Results with multiple comparison correction should be reported.

4. The study recruited 10-15 children of each age between 2 and 16 years old (line 91). When examining the relationship between these endpoints and age (e.g., Figure 1A, Figure 3A), how could we be sure that the observed change was due to age rather than other potential differences between these different groups of children?

5. As the study collected data over 21 days, it will be helpful to know the compliance over time. Was there a decrease in compliance over time? This is important as clinical trials are likely to require a longer time of using wearable and portable devices.

6. Did participants receive any compensation for completing these measures (e.g., temperature, weight, and spirometry measurements)? If so, details should be reported. Strategies on using incentives to improve compliance would be helpful for future clinical trials.
