# Supplementary material for: Towards remote monitoring in pediatric care and clinical trials—Tolerability, repeatability and reference values of candidate digital endpoints derived from physical activity, heart rate and sleep in healthy children
Source: PLoS One. 2021 Jan 7;16(1):e0244877. doi: 10.1371/journal.pone.0244877 (PMC7790377; doi:10.1371/journal.pone.0244877)
Supplement: Attachment — Submitted filename: Kruizinga_review_1.pdf [file pone.0244877.s015.pdf]

Reviewer Comments on
"Towards remote monitoring in pediatric care and clinical trials – Tolerability, repeatability and reference values of candidate digital endpoints derived from physical activity, heart rate and sleep in healthy children."

Manuscript ID: PONE-D-20-30878

November 14, 2020

---

**Summary:** This mauscript presents a study investigating the feasibility of using wearable devices for remote monitoring of children and adolescents with a focus on measures of physical activity, heart rate, and sleep. The manuscript attempts to fill an important gap in the literature by working toward a set of potential clinical endpoints which can be derived from wearable devices and/or collected in a free-living environment using non-invasive technologies.

Overall the manuscript is well written and the study appears to be of high quality. I greatly appreciate the authors' efforts to establish the viability of using wearable devices for remote monitoring of children as a novel contribuiton to the existing literature on wearable devices. However, I have a several concerns as it relates to missing data and/or usable data derived from their wrist-worn device.

My specific major and minor comments are listed below.

**Major Comments:**

1. The authors' choice of 50% estimated wear time as an inclusion criteria for day-level data is not sufficiently justified. For a 24-hour wear-time protocol where sleep, physical activity, and heart rate (all of which have circadian patterns) 50% estimated wear time seems far too low. In adult studies with 24-hour wear time protocol the inclusion criteria is generally much stricter, upwards of 95%.

2. Perhaps even more worrisome is that there appears to be a fundamental disconnect between the estimated wear time and usable data. Using the authors data provided in the supplemental material I created Figures 1, 2, and 3 below. For now focus on Figure 1. From Figure 1 we see that there are a large number of days with missing heart rate data which far exceeds the estimated wear time. For example, there are 60 days with 100% missing heart rate data which have estimated wear time $\geq$ 12 hours, which would lead to the authors including this day in their analysis. Perhaps I'm missing something, but if I'm correct, this discrepency between estimated wear time and usable data necessitates a much more in-depth discussion.

3. I appreciate the inclusion of a variable list in the manuscript, but a more detailed codebook would be helpful. In particular, it would be good to

know what missing (NA) values actually represent. This is particualrly important when it comes to step counts. For example, if we compare the number of mssing values in hourly step counts versus heart rate (see Figure 2) below), we see that there are far more missing step count values that heart rate. These missing values appear to be imputed as 0 when deriving total daily step counts. It would be useful to know whether these values are estimated 0 counts because the individual was wearing the device and not moving, or missing because of non-wear. At a minimum, these missing values and what they mean (or any ambiguity) should be better documented.

4. I have serious concerns about the validity of the data generated by the Steel HR smartwatch. For example, there are a number of days with very little or no usable heart rate data, but have step count data (See Figure 3) and vice versa. The implication would appear to be that different batches of data are contributing to the heart rate and step count results. Have the authors explored sensitivity of their results to this issue? In addition, heart rate measured by PPG has the potential to be quite inaccurate and many algorithms for deriving heart rate from raw PPG signal are not designed to account for movement. Has the Steel HR algorithm been validated in the literature? If not, have the authors explored the higher resolution data (minute, second, sub-second level) for reasonableness?

5. In order for this methods outline in this manuscript to be useful in practice, researchers need to know the exact data processing pipeline involved in creating the analytic dataset. Can the authors describe their data processing pipeline in more detail, perhaps as part of the supplemental meterial? Has this approach been validated before? Or even better, provide the data output from the watches themselves and code used to process the data.

6. The authors should provide code to reproduce their figures and analyses in the supplemental materials rather than upon request.

**Minor Comments:**

1. It's not clear how compliance was calculated. Can the authors expand on how exactly these values were derived?

2. Statistical significance of individual spline coefficients is not particularly helpful for a reader. Given that the authors already present plots of the estimated coefficients with confidence intervals, it may be more useful to present p-values for tests of either no association, linearity, or both.

3. I would encorage the authors to consider using penalized regression splines with a large number of knots rather than low rank unpenalized splines. This can be done easily using the *gamm* package in R which provides an interface between the *mgcv* package for fitting (generalized) additive

models and the lme4 package the authors already use for estimating their mixed effects models. Alternatively, can the authors make a statement regarding sensitivity of their results to choice of the number of spline basis functions?

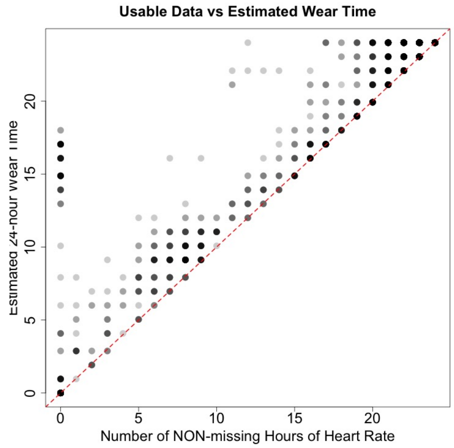


Figure 1: Total daily number of non-missing average hourly heart rate values versus estimated wear time. Points are made semi-transparant due to the overlap of data points.

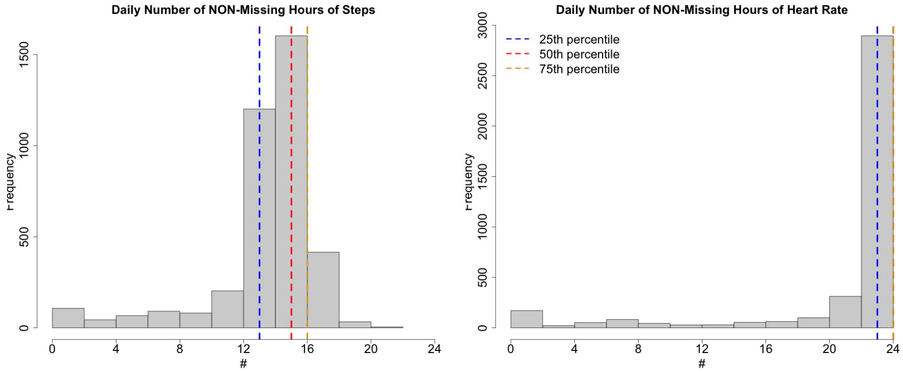


Figure 2: Histograms of the number of the total daily non-missing steps (left panel) and heart rate (right panel). Vertical dashed lines indicate the quartiles of each distribution.

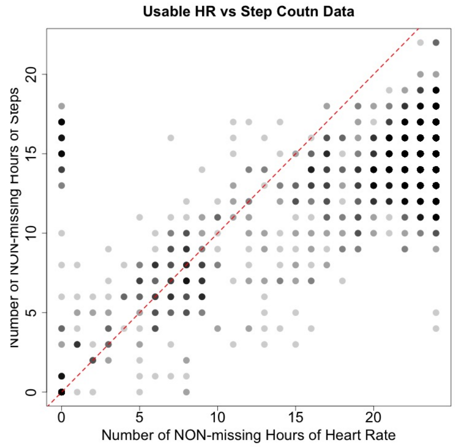


Figure 3: Total daily number of non-missing average hourly heart rate values versus Total daily number of non-missing average hourly step count values. Points are made semi-transparant due to the overlap of data points.
