# Supplementary material for: Towards remote monitoring in pediatric care and clinical trials—Tolerability, repeatability and reference values of candidate digital endpoints derived from physical activity, heart rate and sleep in healthy children
Source: PLoS One. 2021 Jan 7;16(1):e0244877. doi: 10.1371/journal.pone.0244877 (PMC7790377; doi:10.1371/journal.pone.0244877)
Supplement: Attachment — Submitted filename: Kruizinga_review_2.pdf [file pone.0244877.s017.pdf]

Reviewer Comments on Revision 1 of “Towards remote monitoring in pediatric care and clinical trials – Tolerability, repeatability and reference values of candidate digital endpoints derived from physical activity, heart rate and sleep in healthy children.”

Manuscript ID: PONE-D-20-30878

December 10, 2020

Summary: The authors have addressed most of my comments well, though I still have serious reservations about the device used and the methods for choosing days included in the analysis. Given the limitations of the data discussed by the authors in their response regarding estimation of non-wear time, I believe the authors’ approach to estimating non-wear using a combination of non-missing step count or heart rate is reasonable. However, the takeaway message from this seems to be that this device is incompatible with the stated goals of creating a framework for implementing wearable devices in pediatric care trials as wear time cannot be well estimated using the Steel HR device as compared to research grade wearable devices. Further, I remain unconvinced that the 50% estimated wear time during the day inclusion criteria is reasonable, particularly given that the authors are interested in estimating night-time measures.

Major Comments:

1. The issue of wear-time estimation is one of the most challenging methodologic issues related to analyzing wearable device data. The ability to design trials and protocols which maiximize participant/patient compliance is paramount, followed closely by the ability to accurately estimate wear-time. This is particularly true in the context of clinical trials where the interest is in developing clinical endpoints. I understand that the device used in this study substantially limits the authors’ abilities in this area. However, the authors should ackowledge that perhaps this is not the ideal device and/or protocol to use in clinical trials given this critical limitation. In that sense, this study seems to me as more of a “proof of concept” than an actual framework for implementing wearable devices in pediatric trials.

2. The authors claim in their response to my previous Major Comment 1 that the 50% day (06:00-22:00) wear time inclusion critieria is due to the expected lower rate of compliance among children. While I appreciate the additional text devoted to this issue in the discussion section, I would need to see some strong supporting evidence to justify this claim.

   As a comparison, I looked at estimated wear-time compliance between 08:00-20:00 in the NHANES 2003-2006 (a nationally representative sample) accelerometry data for children between the ages of 6 and 15, and

found that the probability of wear compliance during the daytime hours across all 7 days of recorded data (Figure 1) was quite high ($\approx 70\%$) during the waking hours. Note that NHANES 2003-2006 had a "wake-wear" protocol where participants were instructed to remove the device at bedtime and put back on the device upon waking. Given that 1) compliance is lower both with hip-worn devices; 2) compliance tends to be lower with non 24-hour wear protocols; and 3) these children (and their parents) do not have a personal stake in complying with wear-time protocols (as compared to pediatric patients), it seems likely that a patient population using a wrist worn device would have even better compliance, though I'm open to being convinced otherwise. The R code to reproduce Figure 1 is provided at the end of this document.

A sensitivity analysis to choice of threshold may be helpful here.

3. Following up on the wear-time criteria for inclusion of days of accelerometry data, pherhaps I'm missing something, but given that night-time features (heart rate, sleep) are a key component of this analysis, the wear time criteria should include some component of estimated nighttime wear. Or, at a minimum, a discussion of why this potential issue was not considered here.

Minor Comments:

1. Lines 133-134 should probably be "all days with an estimated watch wear time $< 50\%$ between 6AM and 10PM were excluded.."
2. Line 158 should probably be "between 6AM-10PM"
3. Is there a citation for the claim that estimated sleep < 3 hours or > 16 hours are likely invalid? They seem plausible, if rare, values and may indicate underlying health concerns.

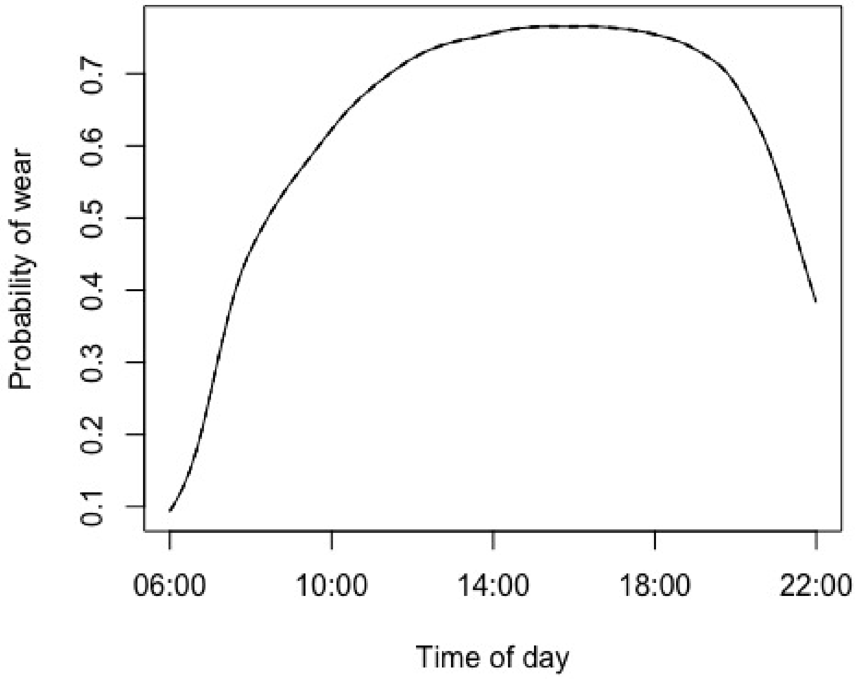

Figure 1: Estimated probability of wearing the hip-worn accelerometer in NHANES 2003-2006 among children aged 6-15.

R code for reproducing Figure 1

```
rm(list=ls())
## Check for packages needed to run analyses/install the rnhanesdata package.
pckgs <- c("devtools","tidyverse","mgcv")
sapply(pckgs, function(x) if(!require(x,character.only=TRUE,quietly=TRUE)) {
install.packages(x)
require(x, character.only=TRUE)
})
rm(list=c("pckgs"))
## Install the rnhanesdata package and dependencies.
## This may take a few minutes because of the size of the data package.
if(!require("rnhanesdata")){
install_github("andrew-leroux/rnhanesdata",build_vignettes = FALSE)
require("rnhanesdata")
}
## load activity count, wear/non-wear flag, demographic/lifestly, and mortality data
data("Flags_C"); data("Flags_D")
data("Covariate_C"); data("Covariate_D")


## combine 2003-2004 and 2005-2006 data
Flags     <- bind_rows(Flags_C, Flags_D)
Covariate <- bind_rows(Covariate_C, Covariate_D)
## merge wear/non-wear flags and covariate data,
## subset to ages 6-16
## and "good" quality data (e.g. device was well calibrated -- PAXCAL and PAXSTAT variables)
df <-
left_join(Flags, Covariate, by="SEQN") %>%
mutate(Age = RIDAGEEX/12) %>%
filter(Age >= 6, Age <= 15, PAXCAL %in% 1, PAXSTAT %in% 1)

## minute columns corresponding to 6AM-10PM
sub_min_cols <- paste0("MIN",(6*60+1):(22*60))
## get wear/non-wear flags for these minutes
sfX <- as.matrix(df[,sub_min_cols])
## impute any missing values as 0
## (there are no missing in this sample, but here for completeness)
sfX[is.na(sfX)] <- 0
## get average wear time for each day
sfX_mn <- rowMeans(sfX, na.rm=TRUE)
## add back into the data matrix
df$sfX_mn <- sfX_mn

## transfor to wide format for fitting a GAM
tind <- (6*60+1):(22*60)
```

```
df_long <-
data.frame("time" = rep(tind, nrow(df)),
"Y" = as.vector(t(sfX)))
## fit the model
##  g(E[Y_ij(t)]) = \beta_0 + f(t)
## note this model assumes independence within subjects and days
## and so standard errors are dramatically underestimated
## also note I'm not incorporating survey weights so technically these results
## are not "nationally representative", but are in-sample estimates
fit <- bam(Y ~ s(time, bs="cr",k=30), method="fREML", discrete=TRUE,
family=binomial(), data=df_long)
## create expit function for plotting on the probability scale
expit <- function(x) 1/(1+exp(-x))
plot(fit, shift=coef(fit)["(Intercept)"], trans=expit,xaxt='n',
xlab="Time of day",ylab="Probability of wear")
xinx <- seq(6,22,by=4)*60+1
xinx_lab <- c("06:00","10:00","14:00","18:00","22:00")
axis(1, at=xinx, labels=xinx_lab)
```
