# Supplementary material for: Towards remote monitoring in pediatric care and clinical trials—Tolerability, repeatability and reference values of candidate digital endpoints derived from physical activity, heart rate and sleep in healthy children
Source: PLoS One. 2021 Jan 7;16(1):e0244877. doi: 10.1371/journal.pone.0244877 (PMC7790377; doi:10.1371/journal.pone.0244877)
Supplement: Attachment — Submitted filename: Response to reviewers comments - 16Dec2020.pdf [file pone.0244877.s018.pdf]

Dear editor and reviewer,

We thank you once more for reading our manuscript and your thoughtful feedback. We have considered your comments carefully and implemented your requests wherever possible. Please refer to our detailed response to your questions below:

Major comments:
1. The issue of wear-time estimation is one of the most challenging methodologic issues related to analyzing wearable device data. The ability to design trials and protocols which maximize participant/patient compliance is paramount, followed closely by the ability to accurately estimate wear-time. This is particularly true in the context of clinical trials where the interest is in developing clinical endpoints. I understand that the device used in this study substantially limits the authors' abilities in this area. However, the authors should acknowledge that perhaps this is not the ideal device and/or protocol to use in clinical trials given this critical limitation. In that sense, this study seems to me as more of a “proof of concept" than an actual framework for implementing wearable devices in pediatric trials.
Response: The estimation of wear time in this study is reliant on several assumptions which may or may not be unacceptable in the context of pivotal clinical trials. However, we feel that the issue is not of a sufficient magnitude to invalidate the results obtained with the device. In total, we aimed to collect 88,200 hours’ (175subjects * 21days * 24h) worth of data. Of these hours, 82,794 (94%) hours had either a valid heart rate measured or had a step count that was higher than 0, indicating that the watch was definitely worn. In addition, 91% of hours had a valid heart rate measured. Note that these numbers regarding missing data include the two 3-year old dropped out subjects.
This amount of missing data is considered inconsequential by many accounts.
(https://pubmed.ncbi.nlm.nih.gov/11688629/)

In the previous rebuttal letter, we conceded that non-wear as defined in the manuscript may be due to either a technical issue or due to actual non-wear. We agree with the reviewer that this issue is good to mention in the interest of transparency, however, this does not mean that the amount of data that is missing is of significant enough size to significantly impact the obtained results. Considering the low frequency of missing data and the (in our view) valid assumption that the data is missing at random, the exact reason of missing data (non-wear or technical limitation) is of no consequence to the estimations obtained during the analysis, and we see no possible reason why the current analysis would lead to skewed or biased results in a particular population or in future clinical trials.

Indeed, considering the wear probability of 70% the reviewer listed from the NHANES study below, we could argue that the steel HR device is at least as suitable for use clinical trials as the medical-grade device used there, since use of the steel HR results in a more complete dataset. We are hesitant to accept the premise of the reviewer that this study merely proves proof-of-concept, and assert that the device is fit-for-purpose for clinical trial use, if proven to have additional value in this context during the clinical validation process, which is extremely important (https://pubmed.ncbi.nlm.nih.gov/32958524/). We have included parts of the reasoning above in the discussion section of the paper.

2. The authors claim in their response to my previous Major Comment 1 that the 50% day (06:00-22:00) wear time inclusion criteria is due to the expected lower rate of compliance among children. While I appreciate the additional text devoted to this issue in the discussion section, I would need to see some strong supporting evidence to justify this claim. As a comparison, I looked at estimated wear-time compliance between 08:00-20:00 in the NHANES 2003-2006 (a nationally representative sample) accelerometry data for children between the ages of 6 and 15, and I found that the probability of wear compliance during the daytime hours across all 7 days of recorded data (Figure 1) was quite high (70%) during the waking hours. Note that NHANES 2003-2006 had a "wake-wear" protocol where

participants were instructed to remove the device at bedtime and put back on the device upon waking. Given that 1) compliance is lower both with hip-worn devices; 2) compliance tends to be lower with non 24-hour wear protocols; and 3) these children (and their parents) do not have a personal stake in complying with wear-time protocols (as compared to pediatric patients), it seems likely that a patient population using a wrist worn device would have even better compliance, though I'm open to being convinced otherwise. The R code to reproduce Figure 1 is provided at the end of this document. A sensitivity analysis to choice of threshold may be helpful here.
Response: We thank the reviewer for this interesting example from the NHANES study. However, the mean compliance (or in this case, probability the watch is worn at a given hour) is not a precise answer to the question: what threshold of wear time are we willing to accept in order to include an given day in our analysis?"

Indeed, the average wear time per hour was higher compared to the NHANES study at around 93% (see figure below), and as such, a higher threshold could be considered. However, we feel that adjusting for wear time during statistical analysis is a valid approach to incorporate the data obtained during the study days where compliance was less than average, and that this is an analysis choice. Whether this approach will be accepted by regulators in pivotal trials is a question to which we do not have an answer, but we see no valid reason why this should not be the case. We have chosen for a cut-off of 50% because we expected compliance to be lower in our cohort (which includes children much younger than the example given from the NHANES study) prior to seeing the results, and based on other publications that adhere to this threshold in the literature, but other investigators may choose higher or lower cutoffs in their statistical analysis plans based on the data presented here. We are hesitant to change this at this point, especially given the fact increasing the threshold to 70% would only exclude an additional 2% of study days which would in no way impact the results obtained in this study. However, we concede that we may need to elaborate on the fact that this was not an arbitrary choice and a threshold which has been reported in pediatrics in the past (e.g. https://www.hindawi.com/journals/bmri/2017/4271483/tab2/, and https://www.ncbi.nlm.nih.gov/pmc/articles/PMC6163401/) and we have incorporated this in the methods section of the paper (line 134-137).

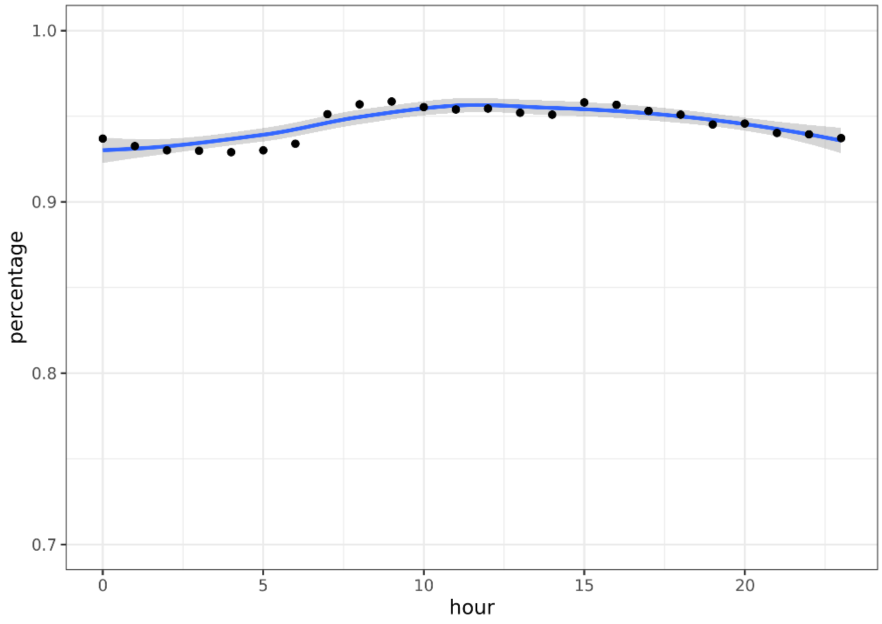

3. Following up on the wear-time criteria for inclusion of days of accelerometry data, perhaps I'm missing something, but given that night-time features (heart rate, sleep) are a key component of this analysis, the wear time criteria

should include some component of estimated nighttime wear. Or, at a minimum, a discussion of why this potential issue was not considered here.

Response: We thank the reviewer for clarifying his point regarding nocturnal wear time and, after discussions within the study team, we have chosen to incorporate a nocturnal wear time threshold for the analysis of nocturnal heart rate and sleep related parameters, only including nights during which 50% of hours between 00AM-5AM contained a valid heart rate measurement. Results regarding nocturnal HR and sleep have been updated throughout the manuscript.

Minor Comments:

1. Lines 133-134 should probably be “all days with an estimated watch wear time < 50% between 6AM and 10PM were excluded."

Response: we thank the reviewer for noticing this oversight and we have addressed this in our correction made with regards to major comment 3.

2. Line 158 should probably be \between 6AM-10PM"

Response: we thank the reviewer for noticing this mistake and have incorporated his suggestion.

3. Is there a citation for the claim that estimated sleep < 3 hours or > 16 hours are likely invalid? They seem plausible, if rare, values and may indicate underlying health concerns.

Response: Based on information obtained from Withings, sleep duration can only be estimated after 3 hours of sleep. Furthermore, published reference values by Iglowstein et al. have total sleep duration have reported a 98th percentile of sleep duration of ~16 hours in 2-year olds, which is why we chose this threshold. Indeed, this threshold could be extended when one would investigate hypersomnic patients, but these values were not expected in the current healthy cohort and must have resulted from device inaccuracies. The limitations of accelerometer-derived sleep duration measurements have been well documented and were already cited in the discussion. We have elaborated on lines 180-181.
