# Supplementary material for: Towards remote monitoring in pediatric care and clinical trials—Tolerability, repeatability and reference values of candidate digital endpoints derived from physical activity, heart rate and sleep in healthy children
Source: PLoS One. 2021 Jan 7;16(1):e0244877. doi: 10.1371/journal.pone.0244877 (PMC7790377; doi:10.1371/journal.pone.0244877)
Supplement: Attachment — Submitted filename: Response to reviewers comments - 17Nov2020.docx [file pone.0244877.s016.docx]

Dear editor and reviewers,

We thank you for reading our manuscript and your thoughtful feedback. We have considered your comments carefully and implemented your requests wherever possible. Please refer to our detailed response to your questions below:

**Journal requirements:**

1. Please ensure that your manuscript meets PLOS ONE's style requirements, including those for file naming.

**Response: we have adapted the manuscript files to comply with the style requirements.**

2.In your Methods section, please provide additional information about the participant recruitment method and the demographic details of your participants. Please ensure you have provided sufficient details to replicate the analyses such as: a) a description of any inclusion/exclusion criteria that were applied to participant recruitment, b) descriptions of where participants were recruited.

**Response: we have included additional details regarding recruitment methods and in- and exclusion criteria to the materials and methods section of the manuscript (p4, line 92-95, tracked changes version).**

3. Please include additional information regarding the survey or questionnaire used in the study and ensure that you have provided sufficient details that others could replicate the analyses. For instance, if you developed a questionnaire as part of this study and it is not under a copyright more restrictive than CC-BY, please include a copy, in both the original language and English, as Supporting Information.

**Response: we have included the end-of-study questionnaire as supplementary material to the manuscript.**

**Reviewer 1**

This study examines the compliance and tolerability of using wearable and portable devices for home monitoring in healthy children between 2 and 16 years old. In addition, the study aims to determine reference values of candidate digital endpoints derived from physical activity, hear rate and sleep data in this population. This work presents an effort towards remote monitoring in pediatric population and holds the potential to provide useful guide for future clinical trials. My major comments are provided below.

1. In the Analysis Section (page 5, line 115), the calculation of the tolerability was discussed. However, little description was provided regarding how the repeatability was quantified in the paper.

**Response: We thank the reviewer for detecting this was not mentioned explicitly and have included additional details regarding this particular analysis to the analysis section (p6, line 149-150, tracked changes version).**

2. In the Results Section, please provide test statistic, p value, and effect size for the findings in the study. For example, when reporting rain duration (Figure 1C) and ambient temperature (Figure 1D) were significantly associated with Daily PA (page 9, line 206), no test statistics were reported to support the conclusion. Similarly, when reporting sleep parameters (lines 253-262), test statistic, p value, effect size and confidence interval should be reported in a consistent way throughout the paper. When reporting a statistically significant correlation between HR and step count (line 248), test statistic and p value should be provided in the text to support the conclusion.

**Response: We agree with the reviewer that additional test statistics should be included in Result section. We have included the requested information above where possible. Due to the sqrt transformation of some of the dependent variables and the inclusion of spline covariates, the absolute effects of continuous variables are impossible to interpret for readers and should be appraised visually with the figures or in the context of the complete model, which is possible with the tables in the supplementary materials. Alternatively, we have provided the ∆AIC and p-value of the likelihood ratio test of the mentioned variables to provide support to our conclusions.**

3. Table 3 includes 7 candidate endpoints, each of which was examined with respect to multiple factors (e.g., age, sex, wear time). The large number of tests conducted could lead to inflated false positives. Results with multiple comparison correction should be reported.

**Response: The reviewer raised a good point with regards to multiple testing, which is generally underused. We agree that multiple testing correction is of high importance in the case of formal hypothesis testing in the context of**

**clinical trials. However, the underlying analysis goal during this study was to identify possible covariates that explain variability. The analysis is exploratory in nature and for this purpose, it is often advised to use Akaike’s Information Criterion (AIC) as a measure of model fit. AIC maximizes the fit/complexity trade-off of a model by balancing the model fit with the number of estimated parameters. We have elaborated on this in the Methods section of the manuscript (p6, line 141-142, tracked changes version)**

4. The study recruited 10-15 children of each age between 2 and 16 years old (line 91). When examining the relationship between these endpoints and age (e.g., Figure 1A, Figure 3A), how could we be sure that the observed change was due to age rather than other potential differences between these different groups of children?

**Response: Of course, as in every study of this type, the observations made in this manuscript are observational and it is formally not possible to determine causal relationships between, for example, age and physical activity. However, we feel that our observations are valid due to the following reasons:**

- **The included children were recruited from the same region in the Netherlands (The Hague area), and one would expect that many unregistered covariates and other sources of variability would be accounted for this way.**
- **Age was included as continuous covariate in the mixed effect models, meaning the information present in the complete cohort is used to determine the overall effect of age, and the effect was not estimated for only the 10-15 per age year.**
- **The age-related effects found in this study are also reported in the literature for both age and heart rate.**

**Still, it cannot be excluded that different effects of age would be found in a completely different cohort, and we have included this possibility in the discussion section of the manuscript (p16, lines 416-418, tracked changes version).**

5. As the study collected data over 21 days, it will be helpful to know the compliance over time. Was there a decrease in compliance over time? This is important as clinical trials are likely to require a longer time of using wearable and portable devices.

**Response: We thank the reviewer for this interesting suggestion. We analyzed the compliance over time for step count, heart rate, sleep, blood pressure, and questionnaire data and found that there was a/no decrease in compliance over time. We felt that the frequency of measurements for temperature, spirometry and weight measurements was too low to adequately estimate the effect of time on compliance. We have included the analysis in the Results section of the manuscript (p8, lines 211-212, tracked changes version). as well as in the supplementary materials.**

6. Did participants receive any compensation for completing these measures (e.g., temperature, weight, and spirometry measurements)? If so, details should be reported. Strategies on using incentives to improve compliance would be helpful for future clinical trials.

**Response: children received a gift certificate worth 20 euros for participation, regardless of their compliance or completion of the study. We have included this information in the Methods section of the manuscript (p4, line 98, tracked changes version).**

**Reviewer 2**

Summary: This manuscript presents a study investigating the feasibility of using wearable devices for remote monitoring of children and adolescents with a focus on measures of physical activity, heart rate, and sleep. The manuscript attempts to fill an important gap in the literature by working toward a set of potential clinical endpoints which can be derived from wearable devices and/or collected in a free-living environment using non-invasive technologies. Overall, the manuscript is well written, and the study appears to be of high quality. I greatly appreciate the authors' efforts to establish the viability of using wearable devices for remote monitoring of children as a novel contribution to the existing literature on wearable devices. However, I have a several concerns as it relates to missing data and/or usable data derived from their wrist-worn device. My specific major and minor comments are listed below.

Major Comments:

1. The authors' choice of 50% estimated wear time as an inclusion criterion for day-level data is not sufficiently justified. For a 24-hour wear-time protocol where sleep, physical activity, and heart rate (all of which have circadian patterns) 50% estimated wear time seems far too low. In adult studies with 24-hour wear time protocol the inclusion criteria are generally much stricter, upwards of 95%.

**Response: We agree with the reviewer that this analysis choice requires additional clarification. We have chosen to use the 50% cutoff for the wear time between 06:00 and 22:00 because these are the hours where steps are expected to be taken by the children. We agree that adjusting for wear time using a linear model disregards the circadian rhythm in physical activity, but feel that in the field of pediatrics, where wear time is expected to be lower compared to adult studies, this is the best way to incorporate as much data as possible in a responsible manner. We have clarified the above further in our manuscript in the discussion section (p16, lines 422-440, tracked changes version), and agree that this choice provides ground for further discussion about how to deal with missing data obtained via remote monitoring in the future.**

2. Perhaps even more worrisome is that there appears to be a fundamental disconnect between the estimated wear time and usable data. Using the authors data provided in the supplemental material I created Figures 1, 2,

and 3 below. For now, focus on Figure 1. From Figure 1 we see that there are a large number of days with missing heart rate data which far exceeds the estimated wear time. For example, there are 60 days with 100% missing heart rate data which have estimated wear time _ 12 hours, which would lead to the authors including this day in their analysis. Perhaps I'm missing something, but if I'm correct, this discrepancy between estimated wear time and usable data necessitates a much more in-depth discussion.

**Response: We greatly appreciate the reviewer’s effort in reviewing our data and providing feedback about our compliance calculation. The way this was performed was the following:**

- **Heart rate and step count data were summarized for each hour**
- **If there was either heart rate OR step count, the compliance for that hour would be graded as ‘worn’.**
- **If there was no heart rate and no step count, the compliance for that hour would be graded as ‘not worn’.**

**If the reviewer would perform the analysis again with these parameters, a perfect correlation between ‘hours worn’ and wear time is expected. We agree this may have been underemphasized in the report and have clarified this further in the Methods section of the manuscript (p5, lines 131-132, tracked changes version). The reason for missing heart rate data for some individual days is, as the author rightly points out in Major comment 4 below, most likely due to the fact that the watch was not worn properly due to inadequate watch position.**

3. I appreciate the inclusion of a variable list in the manuscript, but a more detailed codebook would be helpful. In particular, it would be good to 1 know what missing (NA) values actually represent. This is particularly important when it comes to step counts. For example, if we compare the number of missing values in hourly step counts versus heart rate (see Figure 2) below), we see that there are far more missing step count values that heart rate. These missing values appear to be imputed as 0 when deriving total daily step counts. It would be useful to know whether these values are estimated 0 counts because the individual was wearing the device and not moving or missing because of non-wear. At a minimum, these missing values and what they mean (or any ambiguity) should be better documented.

**Response: We have expanded the codelist with more information. With regarding the step count data that is displayed as NA, this could also be interpreted as ‘0’. However, the reason for zero steps being taken during this hour is unknown by definition and inherent to the field of remote monitoring. Zero steps taken during nocturnal hours are plausible and expected (which is the reason for the majority of the discrepancies observed in the provided figure). However, daytime hours with zero steps are unexpected and could be due to non-wear. We have attempted to solve this issue by looking at HR and step count data simultaneously: if HR data is present, the watch was worn and the ‘0 steps’ are indeed a valid observation. If no HR data is present, it was determined that the watch was not worn and a correction for wear time was applied for the analysis of step count data for these hours only. We have clarified the above further in our manuscript in the discussion section (p16, lines 422-440, tracked changes version).**

4. I have serious concerns about the validity of the data generated by the Steel HR smartwatch. For example, there are a number of days with very little or no usable heart rate data but have step count data (See Figure 3) and vice versa. The implication would appear to be that different batches of data are contributing to the heart rate and step count results. Have the authors explored sensitivity of their results to this issue? In addition, heart rate measured by PPG has the potential to be quite inaccurate and many algorithms for deriving heart rate from raw PPG signal are not designed to account for movement. Has the Steel HR algorithm been validated in the literature? If not, have the authors explored the higher resolution data (minute, second, sub-second level) for reasonableness?

**Response: We agree with the reviewer that these discrepancies warrant much more explanation and discussion and have tried to answer this particular point in our response to the related question 3. Here we would add that the data indeed comes from two different sources (the PPG sensor for heart rate and accelerometer for step count), and the PPG sensor is probably less accurate during movement. Considering the fact that the watch only measures HR every 10 minutes, it is possible that this results in a number of hours without usable HR data. However, the internal algorithm of the watch, developed by Withings, is able to detect inappropriate measurements and discards these. We have appraised the results in our manuscript in the context of applying stricter criteria for, for example, wear time, and the results do not appear to be sensitive to these issues. Model coefficients of course change slightly, but overall effects and estimated means are not significantly different. We have expanded the discussion section to further address these points (p16, lines 422-440, tracked changes version).**

5. In order for this method outline in this manuscript to be useful in practice, researchers need to know the exact data processing pipeline involved in creating the analytic dataset. Can the authors describe their data processing pipeline in more detail, perhaps as part of the supplemental material? Has this approach been validated before? Or even better, provide the data output from the watches themselves and code used to process the data.

**Response: Data is collected using the Withings devices and is transferred to the Withings server based on the protocols employed by Withings, the manufacturer. We have a validated pipeline that requests the data from the Withings server and stores them to a Microsoft Azure Datalake, where the researchers have access to all data. From here, validated scripts are used to process the data in a tabulated data file for statistical analysis, as provided in the supplementary dataset. Since the scripts are highly specific for the particular setup used in our center and the data is stored on the Microsoft Azure server, we feel it is not feasible to include these in our manuscript, but we have included the information above to the Material and Methods section of the manuscript (p7, lines 186-189, tracked changes version).**

6. The authors should provide code to reproduce their figures and analyses in the supplemental materials rather than upon request.

**Response: we have included an R script to the supplementary materials which contains the final statistical analyses presented in the main text of the manuscript.**

Minor Comments:

1. It's not clear how compliance was calculated. Can the authors expand on how exactly these values were derived?

**Response: we have elaborated further on the compliance calculations in the methods section of the manuscript (p5, lines 118-129). In doing so, we have encountered a typographical error in the compliance table for blood pressure measurements (median of 95% instead of 85%, IQR remains 85%-100%).**

2. Statistical significance of individual spline coefficients is not particularly helpful for a reader. Given that the authors already present plots of the estimated coefficients with confidence intervals, it may be more useful to

present p-values for tests of either no association, linearity, or both.

**Response: We agree with the reviewer that p-values for individual spline coefficients are of little scientific value. We have therefore incorporated an additional analysis to the Supplementary Tables to report on the difference in AIC provided by each individual knot for temperature and age in the case of step count per day, and age in the case of heart rate and sleep related parameters. We simultaneously, addressed comment 3 below, and also report the difference in AIC when an additional knot would have been incorporated.**

3. I would encourage the authors to consider using penalized regression splines with a large number of knots rather than low rank unpenalized splines. This can be done easily using the gamm package in R which provides an interface between the mgcv package for fitting (generalized) additive models and the lme4 package the authors already use for estimating their mixed effects models. Alternatively, can the authors make a statement

regarding sensitivity of their results to choose of the number of spline basis functions?

**Response: We thank the reviewer for this thoughtful comment and, have chosen to incorporate the second option and report the value of adding an additional knot as judged by the AIC and likelihood-ratio tests.**
